# Determinants of appropriate complementary feeding practice among mothers having children 6–23 months of age in rural Damot sore district, Southern Ethiopia; a community based cross sectional study

**DOI:** 10.1186/s40795-017-0202-y

**Published:** 2017-11-23

**Authors:** Abate Areja, Dereje Yohannes, Mulugeta Yohannis

**Affiliations:** School of public health, Wolaita Sodo University, Wolaita Sodo, Ethiopia

**Keywords:** Appropriate complementary feeding practice, Infant and young child feeding practice, Damot sore

## Abstract

**Abstract:**

**Background:**

Inappropriate complementary feeding is a major contributor of child malnutrition. Previous studies have described complementary feeding practice using a single indicator but combinations of indicators were needed to better explain the role of complementary feeding practices in child growth and survival. So this study aimed to assess the determinants of appropriate complementary feeding practice among mothers of children 6–23 months in rural Damot sore Woreda, Southern Ethiopia by using a combination of indicators.

**Methods:**

Community based cross sectional survey was carried out in August 2015. The study population comprised of 546 mothers and their children selected using two stage cluster sampling procedure. Data were entered using Epi-data software version 3.1 and analyzed using Statistical Package for the Social Sciences (SPSS) software version 20. Binary and multivariable Logistic regression was used to identify factors associated with appropriate complementary feeding practice and statistical significance was determined at the *p*-value of 0.05.

**Results:**

The response rate was 93.8%. The study showed that level of appropriate complementary feeding practice was 11.4%, 95% CI (8.8, 14.3). Antenatal care follow-up (AOR = 3.2, 95% CI: (1.1, 9.5) and birth order (AOR = 2.4, 95% CI: (1.1, 5.1) were found to have a significant association with appropriate complementary feeding practice.

**Conclusion:**

The study showed only one in eleven mothers practiced appropriate complementary feeding practice for their children aged 6–23 months. Mothers especially during first and second birth order need attention. Moreover, antenatal care follow up should be strengthened and nutrition specific issues should be addressed.

## Background

Malnutrition remains one of the most common causes of morbidity and mortality among children throughout the world. It has been responsible, directly or indirectly, for 60% of the 10.9 million deaths annually among children under five and two-thirds of these deaths, which are often associated with inappropriate feeding practices [1, 2]. Children are most vulnerable to malnutrition in developing countries because of low dietary intakes, lack of appropriate care, and inequitable distribution of food within the household [3, 4]. Over one third of under-five mortality is caused by malnutrition related to inappropriate complementary feeding. Initiate safe and nutritionally adequate complementary foods at 6 month is crucial to achieve optimal growth, development and health [4, 5].

Complementary feeding refers to gradual dietary transition characterized by introduction of solid and semisolid foods to an infant’s diet when breast milk alone becomes insufficient in meeting the nutritional needs of infant. The recommended age range for complementary feeding is generally taken to be 6 to 24 months even though breastfeeding may continue beyond 2 years [1]. Complementary foods are intended to ‟supplement” ongoing breastfeeding and thus facilitate the transition from milk feeding to family foods [6].

The world health organization (WHO) has developed eight core and seven optional indicators to monitor and guide infant and young child feeding practices [7]. Ethiopia is one of the sub Saharan African countries with high level of malnutrition and has launched the national strategy for infant and young child feeding in 2004 to improve the nutritional status of children [8]. WHO recommends a combinations of indicators to measure the level of appropriate complementary feeding, however, most studies conducted so far on complementary practices has used single indicator with narrow age range and thus not adequately quantify the level and determinants of appropriate complementary feeding practices [9]. Among studies conducted using composite indicators, Ethiopia Demographic Health Survey (EDHS) 2011 and study conducted in Abi Adi, Northern Ethiopia showed the level of appropriate complementary feeding to be too low, 4.3% and 10.7% respectively [5, 10]. Furthermore, the information regarding the complementary feeding in the study area is lacking thus this study aimed to assess the prevalence of appropriate complementary feeding practices and associated factors among children aged 6–23 month residing in rural Damot sore district, Southern Ethiopia.

## Methods

### The study area and period

This study was conducted in Damot sore Woreda, Wolaita zone, which is located at 360 km South of Addis Ababa, the capital of Ethiopia. The district has 17 rural and 3 urban kebeles. As to July 2015 the district population is estimated to be 126,850. Among these population 64,690(51%) were females 62,157(49%) males, 19,801(15.6%) under five, 6571(5.2%) under two and 4453(3.5%) children aged 6–23 months. There are five health centers and 20 health posts in Woreda. The study was conducted from August 1–30, 2015.

### Study design, population, and sampling

A community based cross sectional study was conducted. Mothers of children aged 6–23 months living in selected rural households were study populations. The sample size was calculated using single population proportion formula;$n=\left[{{\left(\frac{Z\alpha}{2}\right)}^2}^{\ast }p\left(1-p\right)/{d}^2\right] DE$ by taking proportion of appropriate complementary feeding practice 10.7% from study conducted elsewhere [10]. The following assumptions were used; margin of error = 5%, Z_α_ = 1.96 and design effect (DE) =1.5. Considering 10% contingency to non-responders, a total of 582 mothers were required for study. The study used two stage cluster sampling procedure as follows; first, ten kebeles were selected randomly from 17 kebeles. All kebele has three sub kebeles/ ‘gots’ and all of them were selected making 30 sub kebeles/ ‘gots’. Finally, the total sample was allocated equally to each sub kebeles and data was collected continuously from households having children 6–23 months until desired sample was obtained.

### Data collection procedure and tools

Data were collected by using face-to- face interview during house-to-house visit from mothers who had children aged 6–23 months using structured questionnaire. The questionnaire comprised of information on background characteristics of mother and children, maternal health practice, and child feeding practices. Ten diploma holder data collectors and two BSc. holder supervisors were recruited. For data quality control, the questionnaire was first developed in English and translated to local language, Wolaita language, and then back translated to English by two people, who have good command in both languages, for consistency. Training was given to data collectors and supervisors for 2 days and the questionnaire was pre-tested in 28(5%) of mothers, in the study area, which was not included in actual study to assess the content and approach of the questionnaire and necessary correction was made. All questionnaires were checked on daily bases for completeness. Data was thoroughly checked and cleaned before analysis.

### Operational definitions

**Timely introduction of complementary feeding:** The proportion of children 6–23 months that were introduced to solid and semisolid foods at 6 months of age.

**Minimum dietary diversity:** is the proportion of children 6–23 months of age who receive foods from 4 or more food groups with the food groups consisting; (I) grains, roots and tubers; (II) legumes and nuts; (III) dairy products; (IV) flesh foods; (V) eggs; (VI) vitamin A rich fruits and vegetables; and (vii) other fruits and vegetables during the previous day of study.

**Minimum meal frequency:** is the proportion of breastfed and non-breastfed children 6–23 months of age, who receive solid, semi-solid, or soft foods (but also including milk feeds for non-breastfed children) the minimum number of times or more during the previous day. Minimum is defined as 2 times for breastfed infants 6–8 months, 3 times for breastfed children 9–23 months, 4 times for non-breastfed children 6–23 months.

**Minimum acceptable diet:** is the proportion of children 6–23 months of age who receive both minimum meal frequency and minimum dietary diversity during the previous day of study.

**Appropriate complementary feeding practice:** - In this study defined as if the mother responds correctly for all four indicators, such timely introduction of complementary feeding, minimum dietary diversity, minimum meal frequency and minimum acceptable diet. We declared appropriate; if the mother responds correctly to all four indicators, and inappropriate; if at least one indicator was not correctly fulfilled.

### Data processing and analysis

Data were coded and entered in to Epi-data 3.5.1 statistical software and analysis was made by using statistical package for social science (SPSS) version 20. Descriptive statistics such as frequencies, proportions, means, and standard deviation were used to describe data. Bivariate analysis was made to see the crude significance relation of each independent variable with dependant variable. Finally, independent variables associated during bivariate analysis with P-valueless than 0.25 were entered in to multivariable logistic regression analysis used to determine the strength of association between independent and dependent variables. Odds ratios along with 95% CI were reported and the statistical significance was declared at the *p*-value less than 0.05.

## Results

### Socio-demographic characteristics of mothers

In this study from 582 sampled mothers 546 were participated in the study giving the response rate of 93.81%. From total study participants biological mothers accounted for 531(97.3%) of caregivers while only 15 (2.7%) were other care givers such as grandmothers, siblings and fathers. The median age of mothers was 27 years with interqurtile range (IQR) being 5. Three hundred eighty eight (71.1%) were protestant religion followers. Regarding the educational status of mothers, 329 (60.3%) had attended formal education and 217 (39.7%) did not. The majority of mothers 517 (94.7%) were married and 382 (70%) were housewives by occupation. More than half, 353 (64.7%) of households earned an average monthly income of less than or equal to 999 Ethiopian Birr (ETB). Regarding family size, around half 278(50.9%) have greater than five family and the mean family size of house hold was 5.64 (SD ± 1.87). Majority of mothers 383 (70.1%) had two to four under-five child in the house hold. Husbands of 317 (61.8%) mothers/caregivers had attended formal education and 196 (38.2%) were did not. Four hundred fifty (87.7%) Husbands of mothers/caregivers partners participated on child care regarding complementary feeding practice and 63 (13.9%) not participated **(**Table 1 **)**.

**Table 1:** socio-demographic and economic characteristics of mothers in rural Damot sore district, August 2015

Variables (*N* = 546)	Categories	Frequency(N)	Percentage (%)
Age in years	<20	125	22.9
	20–24	245	44.9
	25–30	127	23.3
	>30	49	9
Marital status	married	510	93.4
	Separated/widowed	36	6.6
Religion	Protestant	388	71.1
	Orthodox	125	22.9
	Catholic	31	5.7
	Muslim	2	0.4
Educational status	No formal education	217	39.7
	Primary education	271	49.6
	Secondary and above	58	10.6
Household head	Father	485	88.8
	Mother	52	9.5
	Grand /siblings	9	1.6
No of under five children	1	163	29.9
	2–4	383	70.1
Support from partner/husband (n = 513)	yes	450	87.7
	No	63	12.3
Educational status of husbands(*n* = 513)	Not attended formal education	196	38.2
	Attended formal education	317	61.8
Monthly income	≤ 999 ETB^a^	353	64.7
	1000–1999 ETB	126	23.1
	2000–2999 ETB	40	7.3
	> = 3000 ETB	27	4.9
	≤ 999 ETB	353	64.7
	1000–1999 ETB	126	23.1

^a^ *ETB* Ethiopian Birr

### Obstetrics and health related variables

Almost all, 466 (87.76%) of mothers attended antenatal care (ANC) follow up at least once during the last pregnancy. About 243(45.76%) had ANC follow up greater than or equal to four times as recommended and 65(12.24%) had not followed ANC at all for their last birth. About 360 (67.8%) of mothers caregivers delivered their last child at home and 171 (32.2%) of mothers delivered at health institution. Four hundred thirteen (77.78%) of mothers/caregivers had received postnatal care (PNC) at least once and 118 (22.22%) had no PNC. Concerning child spacing 75 (13.7%), 250 (45.8%) and 221 (40.5%) give birth of immediate older within 2 year, 2–4 years and ≥4 year respectively. The mean age of immediate older child was (±SD) 48.19 (±25.34) months **(**Table 2 **).**

**Table 2:** The obstetrics and health related characteristics of mothers in rural Damot Sore, district, August 2015

Variables (*N* = 531)	Category	Frequency (N)	Percent (%)
Number of ANC^a^ visit	No ANC session	65	12.24
	≤ three session	223	42.0
	≥ four session	243	45.76
Place of delivery	Home	360	67.8
	Health facility	171	32.2
Post natal care	Yes	431	77.8
	No	118	22.2
Birth spacing with immediate older	<2 years	75	13.7
	2–4 years	250	45.8
	>4 years	221	40.5

^a^ *ANC* antenatal care

### Complementary feeding knowledge and practices

In this study, it was found that the majority 524 (96%) of mothers had ever practiced breastfeeding.

Grains, roots and tubers were the most commonly taken food items by children in previous 24 h preceding survey. Legumes and nuts in the 18–23 months of children’s, dairy products in 6–11 months, vitamin A and other fruits and vegetables in 12–17 months of age group were highly consumed foods, compared to the other groups,24 h preceding survey. Generally, different food groups offered during the past 24 h were uniformly lower in the age group 18–23 months except legumes and nuts, with the lowest rates reported. Also flesh foods and eggs lowest in all groups (8.4%) **(**Table 3 **).**

**Table 3:** Types of food given to children aged 6–23 months by age group (n = 546), in rural Damot Sore district, August 2015

S N^o^	Food groups	Age of children in months											
		6–11 month(211)				12–17 month(188)				18–23 month(147)			
		Yes	(%)	No	(%)	Yes	(%)	No	(%)	Yes	(%)	No	(%)
1	Grains, roots and tubers	185	87.7	26	12.3	182	86.3	6	2.84	147	100	0	0.0
2	Legumes and nuts	45	21.3	166	78.7	59	28	129	61.1	74	50.3	73	49.7
3	Dairy products	169	80.1	42	19.9	162	76.8	26	12.3	128	87.1	19	12.9
4	Egg	19	9	192	91	14	6.64	174	82.5	8	5.44	139	94.6
5	Flesh foods	2	0.95	209	99.1	2	0.95	186	88.2	1	0.68	146	99.3
6	Vitamin A rich foods	21	9.95	190	90	24	11.4	164	77.7	14	9.52	133	90.5
7	Other fruits and vegetable	79	37.4	132	62.6	86	40.8	102	48.3	53	36.1	94	63.9

Four hundred five 74.2%, 95% CI: (70.3%, 78%) of the mothers introduced complementary feeding at 6 months age of the children as per suggested. Twelve (2.3%) mothers introduced complementary feeding early before 6 month, one hundred one (20.3%) mothers initiated late after 6 month and 18 (3.3%) of mothers did not start complementary feeding at all.

**Fig. 1:**
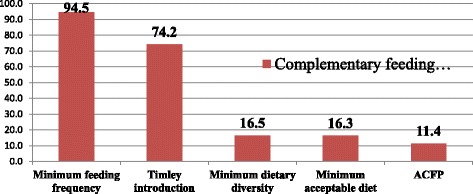
The Appropriate complementary feeding practice by different indicators among mothers in rural Damot Sore district, August 2015

Only 16.5%, 95% CI: (13.4%, 19.6%) mothers offered four or more food groups, and while the rest 79.9% offered less than or equal to three food groups to their child meeting the minimum dietary diversity criteria on the day prior to the study. Five hundred sixteen 94.5%, (95% CI: (92.5%, 96.3%) mothers fed their children to meeting MMF as recommended. Regarding the age-specific prevalence of MMF, 95(87.2%) of breastfed mothers with 6 to 8 month old child, and 388(96.8%) of breastfed mothers with 9 to 23 months had fed their child 2 to 3 times and 3 to 4 times respectively. Thirty four (91.9%) of non-breastfed mothers having 6 to 23 month children fed their child at least four times in 24 h preceding the survey. Only Eighty nine 16.3%, 95% CI: (13.0%, 19.4%) of mothers had practiced the minimum acceptable diet. The level of appropriate complementary feeding practices, by combining the four mentioned indicators, was 62 (11.4%), 95% CI: (8.8%, 14.3%) **(**Fig. 1 **).**

### Factors associated with appropriate complementary feeding practice

Results of bivariate analyses indicated that only antenatal care follow-up of four times and more (COR = 3.4, 95% CI: 1.2, 9.9) was found to be significantly associated with appropriate complementary feeding practice. In the multi-variable logistic regression analysis, two variables were found to be significantly associated with appropriate complementary feeding practice. These were antenatal care follow-up four times and more (AOR = 3.2, 95% CI: (1.1, 9.5) and birth order 3rd or 4th (AOR = 2.4, 95% CI: (1.1, 5.1) were more likely to give appropriate complementary foods to their children **(**Table 4 **)**.

**Table 4:** Factors associated with ACFP among mothers/caregivers of 6–23 months children in rural Damot Sore district, August 2015

Variables	Level	Complementary feeding practices among 6–23 months			
		Appropriate n[%]	Inappropriate n [%]	COR [95%CI]	AOR [95%CI]
Educational status of mothers	No formal education	23[10.6]	194[89.4]	1	
	primary school	29[10.7]	242[89.3]	1.001[0.567, 1.803]	1.078[0.593, 1.959]
	2^ndr^ and above school	10[17.2]	48[82.8]	1.757[0.784, 3938]	1.864[0.783, 4.437]
ANC follow up	No ANC follow up	4[6.15]	61[93.85]	1	1
	≤ 3times	25[11.21]	198[88.79]	1.975[0.657, 5.942]	2.015[0.656, 6.190]
	≥ four times	44[18.11]	199[81.89]	3.413[1.177, 9.896]*	3.235[1.096, 9.546]*
No of under five children	One	24[14.7	139[85.3]	1.568[0.907, 2.711]	1.848[1000, 3.418]
	Two and above	38[9.9]	345[90.1]	1	
Birth order	1st or 2nd	15[10.14]	133[89.86]	1	
	3rd or 4th	32[16.0]	168[84.0]	1.673[0.864, 3.241]	2.403[1.141, 5.059]*
	5th and above	20[10.93]	164[89.07]	1.076[0.524, 2.212]	1.588[0.694, 3.634]
Sex of index child	Male	34[10.9]	277[89.1]	0.907[0.533, 1.544]	0.842[0.486, 1.459]
	Female	28[11.9]	207[88.1]	1	1
Age of index child	6–11 month	20[9.5]	191[90.5]	1	1
	12–17 month	19[10.1]	169[89.9]	1.074[0.554, 2.080]	1.009[0.514, 1.980]
	18–23 month	23[15.6]	124[84.4]	1771[0.934,3.361]	1.733[0.899,3.341]

*p- value <0.05

## Discussion

The overall prevalence of appropriate complementary feeding was 11.4%, (95% CI: 8.8, 14.3). This was comparable to the result from Abiy Adi town, North Ethiopia where appropriate complementary feeding was 10.5% [10]. This might be due to similarities in study setup and indicators used to measure appropriate complementary feeding. The result is higher than the Ethiopia national level and South Ethiopia reported from EDHS 2011 in which prevalence of ACFP was 4.0% and 3.% [5]. However, the result was much lower than the findings from Kohat district Pakistan 18.2% Sirlinka, Zambia and Bangladesh where the prevalence of ACFP was above 20% [8, 11–13]. The disparity might be due to the fact that previous studies were used three indicators to calculate ACFP, whereas this study used four indicators [14, 15].

About 74.2% of mothers had started complementary foods at 6 months of child’s age, as recommended. It was higher than the findings from Deheli India 17.5% [16], United Arab Emirates 17% [17], Mekele 62.8% [18], Ethiopia with national levels 51% [5], Harare 54% [4] and Kohat district Pakistan 69.7% [13]. The result is nearly similar with finding from that of Abiy Adi 80% [10]. This high prevalence might be due to practice group through time, better maternal health care service utilization and extensive effort of health extension workers, health development army and women’s development group in the study area.

Minimum acceptable diet, a combination of minimum meal frequency and minimum dietary diversity, was 16.3% which is higher than Ethiopia national level 4% and SNNPR 2.3%, and Abiy Adi, North Ethiopia 11.9%. But, it was lower than the finding from Sirlanka 68%, Bangladesh 40%, Nepal 32%, Delhi, India19.7% [10–12, 19, 20]. The lower level of the result might be due to socio-cultural differences among the study area. The result is consistent with the findings from Tanzania (16.3% Vs 15.9%) [21].

MDD was observed only 16.5% of caregivers were fed their young child with food group four and more from seven food groups namely grains roots and tubers, legumes and nuts, dairy products, flesh foods, vitamin A reached foods, eggs and other fruits vegetables. Ergib Mekibib et al. also reported from northern Ethiopia that 17.8% of mothers fed their child from four and more food groups and the result of this study is lower than findings from Kamba Woreda SNNPR was 23.3%, Delhi, India 32.6% [19, 22].

The prevalence of MMF was higher than national level 4.3% and SNNPR 2.5%. Moreover, the level of minimum meal frequency in the study found to be 94.5%. The result was much higher than the national level 47.9% and SNNPR 48.9% [5].

The study revealed that ANC follow up and birth orders are factors significantly associated with appropriate complementary feeding practices. Mothers who followed antenatal care service were 3.2 times more likely to practice appropriate complementary feeding than those who did not follow the ANC service. This result is similar with the findings in Sirlinka, Nepal; Harare [11, 20, 23] ANC contacts were a significant predictor of appropriate complementary feeding practice. This might be due to the result of information and counseling that the mothers received from health care providers during their antenatal care. Birth order were found to be the predictors ACFP and this result is congruent with the findings from Kemba [22]. This might indicate the previous feeding experience that could enhance appropriate feeding practice.

In this study there were no association of index child age and, appropriate complementary feeding. This result were different findings from Ethiopia and Zambia, Tanzania; Indonesia [8, 21, 24]. This might be due to the difference in socio cultural understanding of mothers about young children can be able to digest all foods. In this study there was no association between education of caregiver’s and appropriate complementary feeding practice. This was disagreed with the findings from Indonesia, Ethiopia and Zambia, Kenya, Nepal and Sirlanka where maternal education is the predictors of appropriate complementary feeding [8, 10, 11, 20, 24, 25]. The possible explanations can be the difference in norms and cultures with geographical difference regarding female education.

### Limitations of this study

The possible limitation of this study was that infant feeding practices are age-specific with narrow age ranges and characteristically assessed by mothers report on recall; this may lead to recall bias.

## Conclusion

Based on the findings, only one out of nine mothers fed complementary foods appropriately to their children. Although the study showed the high prevalence of minimum meal frequency and timely introduction of complementary feeding, the prevalence of minimum dietary diversity and minimum acceptable diet was low which contributed to the lower prevalence of appropriate complementary feeding. The study also identified mothers who have ANC four and more times and had birth order three or four were factors significantly associated with appropriate complementary feeding practice. Appropriate complementary feeding practice should not be measured by considering only one or two indicators, but by using combinations of core indicators. Capacity building should be strengthen for all health care providers including health extension workers, health development army and women’s support group to focus on dietary diversity and feeding frequency. Health care workers should encourage the women to attend and compliance ANC giving attention to premi gravidas.

## Abbreviations

ACFP: Appropriate complementary feeding practices
ANC: Antenatal care
AOR: Adjusted odds ratio
COR: Crude odds ratio
EDHS: Ethiopia Demographic Health Survey
PNC: Postnatal Care
SNNPR: Southern Nations Nationalities and Peoples Region
